# A new journal for a new age

**DOI:** 10.1093/lifemeta/loac001

**Published:** 2022-04-29

**Authors:** Peng Li, John R Speakman

**Affiliations:** State Key Laboratory of Membrane Biology and Tsinghua-Peking Center for Life Sciences, Beijing Advanced Innovation Center for Structural Biology, School of Life Sciences, Tsinghua University, Beijing, China; Shanghai Qi Zhi Institute, Shanghai, China; Shanghai Key Laboratory of Metabolic Remodeling and Health, Institute of Metabolism and Integrative Biology, Fudan University, Shanghai, China; State Key Laboratory of Molecular Developmental Biology, Institute of Genetics and Developmental Biology, Chinese Academy of Sciences, Beijing, China; University of Chinese Academy of Sciences, Beijing, China; Institute of Biological and Environmental Sciences, University of Aberdeen, Aberdeen, UK; Shenzhen Key Laboratory of Metabolic Health, Center for Energy Metabolism and Reproduction, Shenzhen Institutes of Advanced Technology, Chinese Academy of Sciences, Shenzhen, China

Exactly one hundred years ago, the world was emerging from the ravages of the Spanish flu pandemic that killed 30 million people. Today, we are also emerging from a similar pandemic caused by coronavirus disease (COVID-19). However, the world that survivors of the flu pandemic inherited was very different from the world that will be left after COVID-19 is finally over. Life, of course, has changed enormously in these last 100 years with the advent of air travel, cars, computers, and the internet. But not only healthy life has changed, but what has also changed is the reasons why we get ill and die. After the flu pandemic, the major killers were still infectious diseases and undernutrition. Nowadays, the main health problems are due to over-nutrition and non-communicable diseases such as obesity, Type 2 diabetes, cardiovascular diseases, cancer, and neurological diseases. Moreover, because more people live into old age nowadays, we face many other issues connected with dementia and other disorders of aging.

Metabolism provides a bridge between all these problems. It is fundamental to life as it covers all biochemical reactions and is an interdisciplinary area covering biochemistry, molecular and cell biology, and organismal physiology and pathology. Metabolism also interfaces with developmental biology, cardiovascular biology, immunology, and neurobiology. Not surprisingly, metabolism is also of immense interest for drug discovery. As such, research in the field of metabolism holds an unrivaled position in biomedical research.

In recent years, there has been a profound growth in metabolic research, a field with a long and rich history. A proliferation of new technologies has further allowed scientists to delve more deeply into metabolic processes than ever before. The ‘renaissance of metabolism’ has also attracted brilliant minds in other fields, while nourishing new talents within the discipline.

We are, therefore, excited to announce the inauguration of *Life Metabolism*, a new international journal for metabolism-related studies, under the partnership with Oxford University Press (OUP) and Higher Education Press of China. *Life Metabolism* will be a new platform for the publication of high-quality studies related to metabolic research, for discussions of broad relevance to research community and for public education. All areas of metabolism will be covered by the journal, including but not limited to nutrient sensing and signaling, energy metabolism, immunometabolism, neurometabolism, exercise metabolism, circadian rhythms, aging, microbiome effects, obesity and metabolic syndrome, cancer metabolism, metabolism-related clinical studies, advanced technologies, and advances in translational studies.

Our Editorial Board currently consists of a panel of 78 distinguished international experts in metabolic research. The journal is committed to provide a fair, fast, and transparent review process. In the first 3 years, there will be NO publication charges for publishing in *Life Metabolism*, and Open Access fees will be waived.

We appreciate your support of and interest in *Life Metabolism* and also look forward to serving the international metabolism community for many years to come. Please follow our activities on our website (https://academic.oup.com/lifemeta) or on Twitter (@LifeMetabolism and @johnspeakman4).

